# The Lady House Surgeon

**Published:** 1916-06-24

**Authors:** 


### THE LADY HOUSE SURGEON.

### By an Old-Time Matron.

It was the summer of 1915. The board of a small general hospital had lost the services of both its house surgeons. A dear old *locum* of seventy had offered his services as a substitute until the board was suited, but he was staggering a little under his many claims, and found the night work very trying and looked out anxiously for relief. The medical board had been, and were, decidedly averse to women having any say in any matter. But no man answered advertisements either in the *British Medical Journal*, the *Lancet*, or THE HOSPITAL.

THE WOMEN APPLICANTS.

Again advertisements were inserted, and once more ladies applied. There was almost revulsion at considering the testimonials. Still the need was great, for even the honorary staff had diminished. Two ward sisters had been called up, and had gone at a few hours' notice. The "time-up" probationers, instead of proudly "holding on" for sister's summer holiday duties, were feverishly rejoiced at being accepted for war work elsewhere! There was nothing for it but to consider these ladies' applications, and to request the one that seemed the least objectionable to appear before the board. Miss York was therefore asked to appear. "Her testimonials were good. She might at least be able to give an anæsthetic."

THE APPOINTMENT MADE.

So one day she passed the criticisms and questionings of the board in a quiet, unassuming manner hardly expected—she even scored once or twice when slightly harried by a crusty old surgeon—and when she thanked the board for appointing her, it certainly seemed more pleasant than the nonchalant but nervous manner of the usual house surgeon. It was a long time since the Nurses' Home had enjoyed such an excitement, although hope and fear had seen what was coming, and it must be told with sorrow that disgust filled their souls when the news was told. The sisters' tea-table was an angry one, despite little Sister Casualty saying, "She ought to have a chance." Sister Jones asserted, "I won't let my nurses carry screens for her," and the probationers saying less, yet felt their grievance was great. Matron's experience of some youthful house surgeons made her less perturbed. Anxiety was too frequent a visitor to cause apprehension to be formidable. It had been rather restful with the old *locum*, although his health was not of great account. Perhaps Miss York might do!

THE LADY H.S. TAKES OFFICE.

She came one afternoon. A neat figure dressed just right for her work, a pleasant face, but one with a firm cleverness that somehow made rebels a little uncertain. Before a week was gone the personality of Dr. York was admitted by all. The title was her one vanity; she had worked so hard to gain it. The honorary staff were the first to give way, and to be grateful for this womanly house surgeon who yet was so thorough and capable. She was the keenest in the hospital; ready at a single call to turn out at night, appearing happily bright where she was needed. She had an individual interest in all. It was not long before in- and out-patients loved and trusted her. No scandal-monger; she did not discuss others, and her rare praise was felt to be worth having. The truthful reports and accurate writing up of history sheets, the attention to detail, and avoidance of unnecessary interference were lessons to be remembered. The probationers found her a good ally at tennis when off duty; even the sisters took hints, and left off blaming the nurses when things went wrong. Could it be possible to say the hospital was the better for a *lady* house surgeon, who found time to hold ambulance classes for the young men from the town, and held them, as one said, "a lot better than the doctors"?

HER HUMANITY APPARENT.

Being human she occasionally made mistakes, such as allowing a patient up before informing the sister, or altering treatment through a probationer, or ordering a new drug without consulting the honorary. She certainly was rather stupid over seniority, and could not understand at first why a dull, incapable nurse should take precedence of a brighter, more gifted woman just because she had entered upon her training a little sooner; and she could not help showing preference for the earnest, willing spirits, and now and then some sharpness was felt when an order was not understood, although little was said. Women are holding many posts now, but few are so commendable as those like Miss York, who have been able to prove the value of lady house surgeons.

